# C-reactive protein velocity to distinguish febrile bacterial infections from non-bacterial febrile illnesses in the emergency department

**DOI:** 10.1186/cc7775

**Published:** 2009-04-08

**Authors:** Yael Paran, Doron Yablecovitch, Guy Choshen, Ina Zeitlin, Ori Rogowski, Ronen Ben-Ami, Michal Katzir, Hila Saranga, Tovit Rosenzweig, Dan Justo, Yaffa Orbach, Pinhas Halpern, Shlomo Berliner

**Affiliations:** 1 Department of Internal Medicine "D", "E" and "H", Tel-Aviv Sourasky Medical Center, 6 Weitzman Street, Tel-Aviv 64239, Israel; 2 Department of Infectious Diseases, Tel-Aviv Sourasky Medical Center, 6 Weitzman Street, Tel-Aviv 64239, Israel; 3 Department of Molecular Biology, Ariel University Center of Semaria, Ariel 40700, Israel; 4 General Laboratory, Schneider Children's Medical Center, 14 Kaplan Street, Petach-Tikva 49202, Israel; 5 Department of Emergency Medicine, Tel-Aviv Sourasky Medical Center, 6 Weitzman Street, Tel-Aviv 64239, Israel

## Abstract

**Introduction:**

C-reactive protein (CRP) is a real-time and low-cost biomarker to distinguish febrile bacterial infections from non-bacterial febrile illnesses. We hypothesised that measuring the velocity of the biomarker instead of its absolute serum concentration could enhance its ability to differentiate between these two conditions.

**Methods:**

We prospectively recruited adult patients (age ≥ 18 years) who presented to the emergency department with fever. We recorded their data regarding the onset of fever and accompanying symptoms. CRP measurements were obtained upon admission. CRP velocity (CRPv) was defined as the ratio between CRP on admission and the number of hours since the onset of fever. Patients were diagnosed by clinical symptoms, blood cultures and imaging studies, and the diagnoses were confirmed by an infectious disease specialist. The efficacy of CRPv as a diagnostic marker was evaluated by using receiver operator curves (ROC). Excluded were patients who did not know the time fever started with certainty, patients with malignancy, patients with HIV infection and patients who had been using antibiotics upon presentation.

**Results:**

Of 178 eligible patients, 108 (60.7%) had febrile bacterial infections (mean CRP: 63.77 mg/L, mean CRPv: 3.61 mg/L/hour) and 70 (39.3%) had non-bacterial febrile illnesses (mean CRP: 23.2 mg/L, mean CRPv: 0.41 mg/L/hour). The area under the curve for CRP and CRPv were 0.783 (95% confidence interval (CI) = 0.717 to 0.850) and 0.871 (95% CI = 0.817 to 0.924), respectively. In a 122-patient subgroup with a CRP level of less than 100 mg/L, the area under the curve increased from 0.689 (95% CI = 0.0595 to 0.782) to 0.842 (95% CI = 0.77 to 0.914) by using the CRPv measurements.

**Conclusions:**

CRPv improved differentiation between febrile bacterial infections and non-bacterial febrile illnesses compared with CRP alone, and could identify individuals who need prompt therapeutic intervention.

## Introduction

There are many lines of evidence to support the usefulness of C-reactive protein (CRP) as a real-time and low-cost biomarker for differentiating between acute bacterial and non-bacterial infections [1-8]. We hypothesized that using the velocity of the biomarker, by integrating the time of fever onset with its absolute serum concentration, would further enhance differentiation. This concept is not new; the sensitivity of a biomarker in some cases depends on the time lapsed from the onset of symptoms to presentation. For example, when evaluating patients with acute coronary syndromes, the time of onset of chest pain is crucial for correctly interpreting the levels of cardiac enzymes such as troponin [9]. In the current work, we adopted a similar approach in the context of acute febrile diseases in the department of emergency medicine. To the best of our knowledge, no study has previously evaluated the velocity of change in CRP in the setting of an acute febrile disease.

We defined 'CRP velocity' (CRPv) as the rate at which CRP changes over time. The rate is defined as the CRP value at the time a patient presents with an acute febrile disease divided into the number of hours since the patient first noticed having fever. The encouraging results described below support the need for further evaluation of this concept in the setting of acute infections and an impending cytokine storm.

## Materials and methods

### Patients

This was a prospective study performed with the approval of the local ethics committee. In the study, adult patients (aged ≥ 18 years) who were admitted to the department of emergency medicine at the Tel-Aviv Sourasky Medical Center, Israel, with a history of an acute (< two weeks' duration) febrile condition were recruited upon presentation and gave informed consent of participation in the study. Included were only patients presenting to the department of emergency medicine with an oral temperature of 38.0°C or above that could specify the exact time of fever onset, defined as home oral temperature of 38.0°C or above.

Exclusion criteria were an underlying malignancy, HIV infection or use of antibiotics. Recruitment into the study took place upon the patient's presentation to the emergency room and after written informed consent had been obtained according to the instructions of the local ethics committee and before any administration of antibiotic treatment. Bacterial cultures, laboratory tests and imaging studies were preformed at the discretion of the attending physician in the emergency room.

At the end of their stay in the emergency room, the patients were either discharged or admitted to hospital. The relevant clinical, laboratory and imaging data were collected for study entrants. Special attention was given to the time of fever onset. Patients were asked to specify in as much precision as possible the exact time they first noticed they were febrile. Follow-up was obtained for all participants. Individuals who were discharged from the department of emergency medicine were contacted by telephone by one of the investigators (YP) between 5 and 10 days after discharge. Data were obtained on the current status of the patient and, specifically, whether the fever had resolved with or without the administration of antibiotics.

A specialist in infectious diseases (RB or MK) that was blinded to the results of the patient's CRP findings retrospectively reviewed all the medical records and classified the patient into one of two diagnostic categories: definite or probable acute bacterial infections, or non-bacterial febrile illnesses. The definite or probable acute bacterial infections group was based on either a positive bacterial culture from a relevant clinical focus or on the diagnosis of infections which were most probably due to a bacterial etiology. These diagnoses were defined according to standard clinical criteria; for example, an obvious cellulites or clinical criteria consisting with pneumonia, together with a pulmonary infiltrate on chest x-ray confirmed by a radiologist. Patients were categorized as being in the non-bacterial febrile illnesses group if the fever resolved without any antibiotic treatment. Also included were individuals who had both a clinical picture of viral infection and a positive serology consistent with viral infection (e.g., mononucleosis-like disease and a positive immunoglobulin (Ig) M for Epstein Barr virus), as well as patients with non-infectious febrile conditions (e.g., exacerbations of autoimmune disorders). The definitions of bacterial as well as viral infections were consistent with 17^th ^edition of Harrison's textbook of Internal Medicine [10]. Patients who did not clearly fit either category were dropped.

### Methods

We defined a new parameter 'CRPv' to represent the value of the CRP of a patient presenting with an acute febrile disease divided into the number of hours since the patient first noticed having a fever. This reflects the rate at which CRP changes over time. Contrary to the velocities that are based on two measurements of a biomarker, we used a single measurement of CRP to calculate velocity, because we were looking for a marker to distinguish febrile bacterial infections from non-bacterial febrile illnesses upon presentation.

### Laboratory methods

Blood samples were drawn immediately after study recruitment for complete blood count and measurement of CRP levels. CRP was evaluated by an immunoturbidimetric assay on the ADVIA 1650 chemistry system (Bayer, Leverkusen, Germany) using the Bayer ADVIA kit for wide-range CRP. Complete blood count was evaluated by coulter STKS system (Beckman Coulter, Nyon, Switzerland). CRP, white blood count (WBC) and neutrophil count cut-offs in the local laboratory were 5.0 mg/l, 11000/ml^3 ^and 6000/ml^3^, respectively. Samples for procalcitonin (PCT) were analyzed in a subgroup of 48 patients by using the LIAISON BRAHMS 2-site immunoluminometric assay (Liaison Brahms PCT; Brahms Diagnostics, Berlin, Germany), run on the Diasorin Liaison instrument. The measuring range in this assay was 0.1 to 500 ng/ml.

### Statistical analysis

All data were summarized and displayed as mean ± standard deviation (SD) for the normally distributed continuous variables, geometrical mean plus the quartiles for non-normally distributed continuous variables and number of patients plus the percentage in each group for categorical variables. CRP concentrations displayed irregular distribution, so we used a logarithmic transformation that converted the distribution to a normal one for all statistical procedures. Therefore, all results of those variables are expressed as back-transformed geometrical mean. The One-Way Kolmogorov-Smirnov test was used to assess the distributions.

For comparing continuous variables, an independent sample Student's t-test analysis was performed for the normally distributed variables, while the Mann-Whitney analysis was used for non-normally distributed variables to compare the various parameters between the bacterial and the non-bacterial groups. The chi-squared test was used to assess the overall significance between the groups for all categorical variables. Furthermore, in order to evaluate the performance of classification schemes of the different variables and to compare the classification of the two groups of patients (bacterial and non-bacterial), we used a receiver operated characteristic curve (ROC) analysis. We calculated the area under the curve (AUC) to compare the classifiers and the asymptotic statistical significance to reject the hypothesis that the curve is similar to the reference line, which is a random classifier. Finally, we conducted further analysis using a CRP value of 100 mg/L or lower, because higher values above 100 mg/L have been shown to indicate bacterial infection [2,11,12]. All the above analyses were considered significant at a *P *value less than 0.05 (two-tailed). The SPSS statistical package was used to perform all statistical evaluations (SSPS Inc., Chicago, IL, USA).

## Results

A total of 215 patients met the inclusion criteria for the current study. After reviewing their data, the infectious disease specialists categorized 22 patients (10.2%) as inconclusive (20 of them received antibiotics even though the fever at the time of admission could have been of viral origin). They categorized 15 patients (7.0%) as most probably viral, but they, too, were treated with antibiotics after admission and excluded. Thus, a total of 178 patients remained for analysis of which 108 (60.7%) were classified as having a bacterial infection and 70 (39.3%) as having a non-bacterial febrile illness. Half of the patients in the bacterial infection group were men and half were women, while in the non-bacterial febrile illness group only 27 patients (38.6%) were women (*P *= 0.135). Mean age of patients with bacterial infection was higher relative to patients with non-bacterial febrile illness (54.6 ± 23.4 vs. 33.1 ± 16.1 years; *P *< 0.001), as were the prevalence of co-morbidities such as ischemic heart disease (19.4 vs. 2.9%; *P *= 0.001) and diabetes mellitus (21.3 vs. 4.3%; *P *= 0.002). The mean body mass index (BMI) was also higher for the bacterial group (25.4 ± 4.5 vs. 23.5 ± 4.1; *P *= 0.003). Steroid usage, on the other hand, which represented immunodeficiency, was no more prevalent among patients with bacterial infection compared with patients with non-bacterial febrile illness (Table 1).

**Table 1:** Demographic characteristic, medications on admission and co-morbidities in the bacterial and non-bacterial groups

		**Bacterial** **n = 108**	**Non-bacterial** **n = 70**	***P *value**
Age, years	Mean ± SD	54.6 ± 23.4	33.1 ± 16.1	< 0.001
Women	n (%)	54 (50.0%)	27 (38.6%)	0.135
Body mass index, kg/m^2^	Mean ± SD	25.4 ± 4.5	23.5 ± 4.1	0.003
Ischemic heart disease	n (%)	21 (19.4%)	2 (2.9%)	0.001
Diabetes mellitus	n (%)	23 (21.3%)	3 (4.3%)	0.002
Steroids usage	n (%)	2 (1.9%)	1 (1.4%)	0.83

SD = standard deviation.

The diagnosed infections were classified according to the site of infection and are listed in Table 2, and the pathogens isolated in cultures or demonstrated by serology are listed in Table 3. As expected, the patients with bacterial infection had significantly higher CRP levels than those with non-bacterial infection (geometrical mean of 63.77 mg/L vs. 15.23 mg/L, respectively, *P *< 0.001). The febrile illness CRPv was also significantly higher in the bacterial compared with the non-bacterial group (3.61 mg/L/hour vs. 0.41 mg/L/hour, respectively, *P *< 0.001).

**Table 2:** Infectious diagnosis according to site of infection

**Bacterial infection (n = 108)**	**Non-bacterial febrile illness (n = 70)**
Pneumonia (n = 38)*	Unspecified viral infection (n = 19)
Urinary tract infection (n = 34)*	URTI/bronchitis (n = 16)
Skin, soft tissue (n = 13)	Gastroenteritis/colitis (n = 16)
Pharyngitis (n = 12), sinusitis (n = 1)*	EBV, CMV (n = 9), herpes zoster (n = 1)
Gastroenteritis/colitis (n = 10)	Viral meningitis (n = 3)
Abdominal infection (n = 2)	Hepatitis (n = 2)
	Autoimmune disorders (n = 4)

*Two patients had concomitant infections: one had pneumonia and a urinary tract infection, and one had pneumonia and sinusitis. CMV = cytomegalovirus; EBV = Epstein Barr virus; URTI = upper respiratory tract infection.

**Table 3:** Pathogens isolated in cultures or demonstrated by serology

**Blood cultures (n)**	**Urine cultures (n)**	**Soft tissue and skin abscess cultures (n)**	**Stool cultures (n)**	**Throat cultures (n)**	**Serology (n)**
*Escherichia Coli *(7)	*Escherichia coli *(12)	*Streptococcus pyogenes *(3)	*Shigella sonnei *(2)	Group C Streptococcus (2)	*Mycoplasma pneumonia *(1)
*Acinetobacter baumannii *(1)	*Pseudomonas *aeruginosa (1)	Methicillin-sensitive *Staphylococcus aureus *(1)	*Campylobacter *jejuni (2)	*Streptococcus pyogenes *(3)	Cytomegalovirus (4)
*Klebsiella pneumoniae *(1)	*Enterococcus faecalis *(2)	*Pseudomonas *aeruginosa (1)			Epstein-Barr virus (5)
Methicillin-sensitive *Staphylococcus aureus *(1)	*Klebsiella oxytoca *(1)				Coxsackievirus B4 enterovirus (1)
*Enterococcus *faecalis (1)	*Klebsiella pneumoniae *(1)				Hepatitis B virus (1)
					Herpes simplex virus (1)

Eight patients had two positive cultures at the same time.

**Figure 1:**
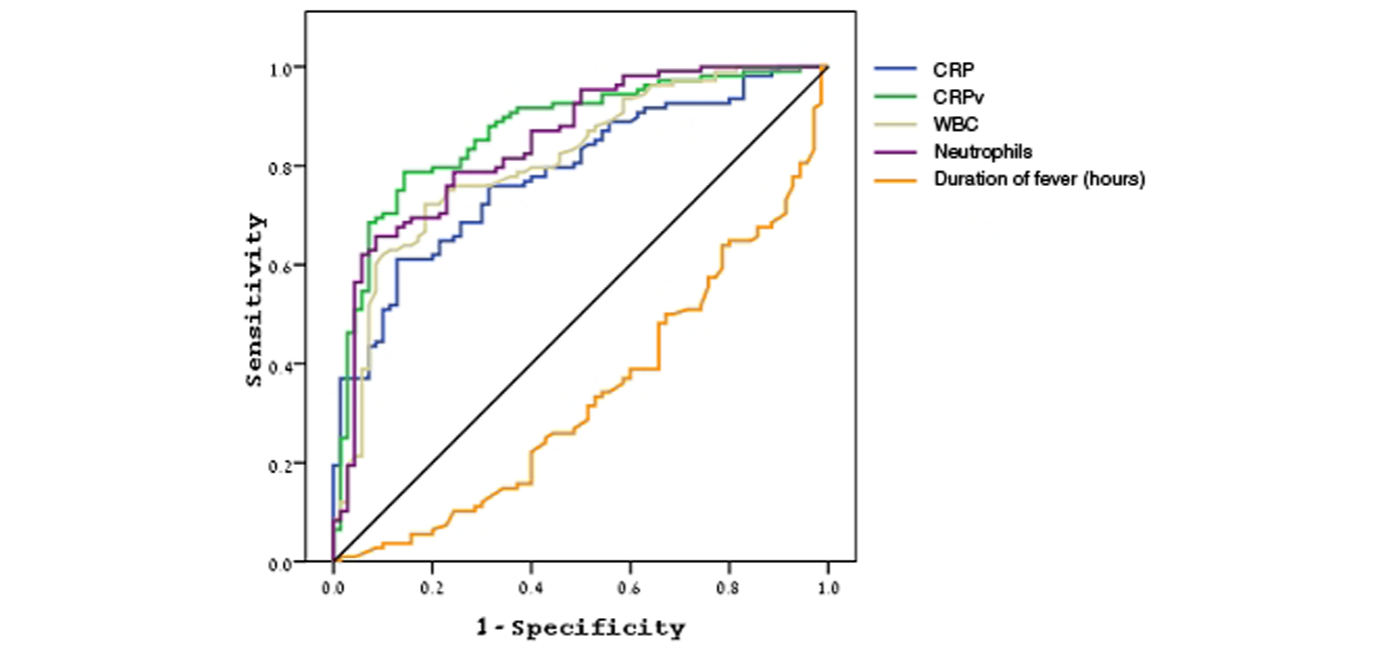
Receiver operator curve for C-reactive protein and C-reactive protein velocity for the diagnosis of bacterial infection. CRP = C-reactive protein; CRPv = C-reactive protein velocity; WBC = white blood cells.

The CRP and CRPv geometric means, median and interquartile ranges, and the mean WBC and neutrophil counts and their SD in the two groups are shown in Table 4. The efficacy of CRP measurements and CRPv in differentiating between bacterial and non-bacterial febrile illness was evaluated by ROC analysis. The AUC for CRP and for CRPv were 0.783 (95% confidence interval (CI) = 0.717 to 0.850) and 0.871 (95% CI = 0.817 to 0.924), respectively (Figure 1).

**Table 4:** Comparison of different parameters between patients with bacterial infection and those with non-bacterial infection

	**Variable**	**Duration of fever (hours)**	**CRP (mg/L)**	**CRP-velocity (mg/:/hour)**	**WBC (10^3^/ml^3^)**	**Neutrophils (10^3^/ml^3^)**
Bacterial (n = 108)	Mean	36.5	63.77	3.61	13.94	11.74
	Interquartile range	6.0 to 46.8	37.4 to 156.0	1.97 to 7.41	9.85 to 17.55	7.47 to 15.41

Non-bacterial (n = 70)	Mean	68.7	15.2	0.41	8.35	5.77
	Interquartile range	17.8 to 90	9.8 to 51.6	0.18 to 1.49	5.88 to 9.75	3.44 to 7.31

*P *value		< 0.001	< 0.001	< 0.001	< 0.001	< 0.001

CRP = C-reactive protein; WBC = white blood cells.

**Figure 2:**
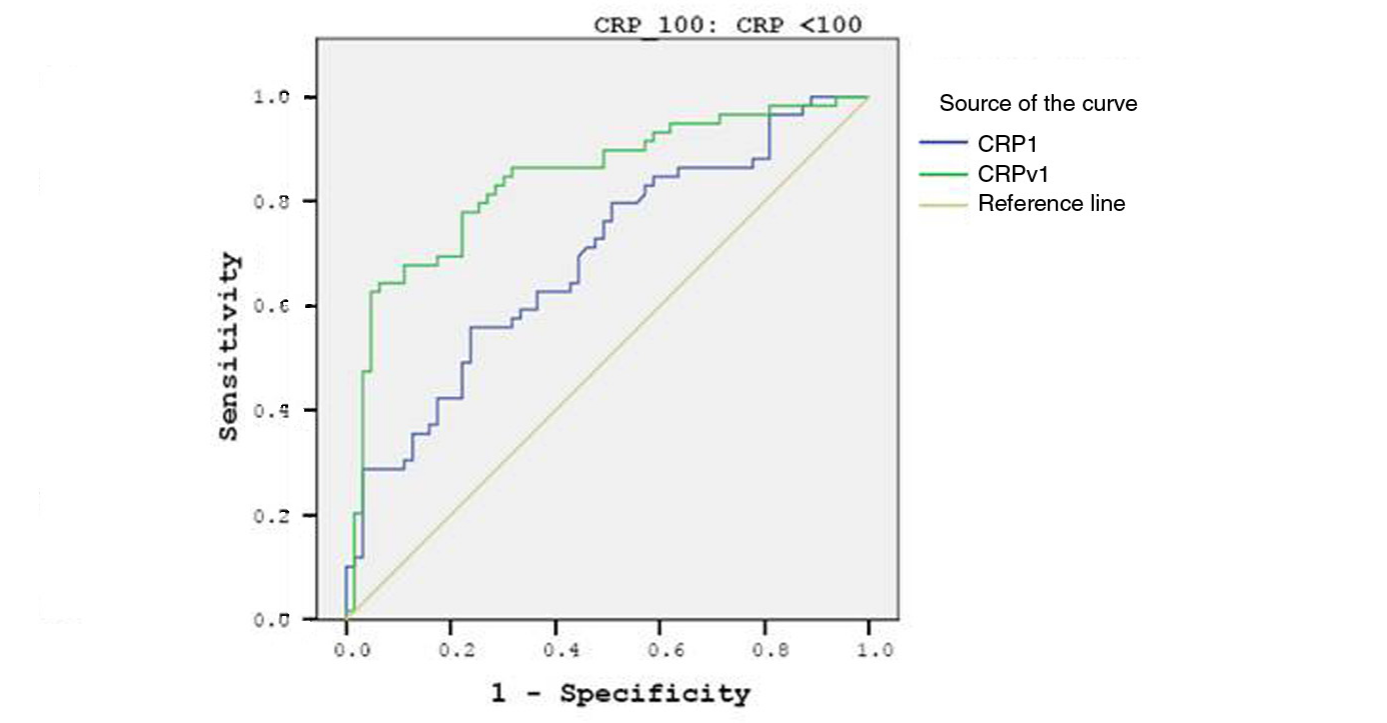
Receiver operator curve for C-reactive protein and C-reactive protein velocity for the diagnosis of bacterial infection for patients with C-reactive protein less than 100 mg/L. CRP = C-reactive protein; CRPv = C-reactive protein velocity.

There were 122 patients with a CRP concentration of less than 100 mg/L, of whom 59 had bacterial and 63 had non-bacterial febrile illness. An ancillary analysis was conducted for this subgroup of patients. The mean, median and interquartile ranges are shown in Table 5. The AUC for CRP and CRPv for this subgroup of patients were 0.689 (95% CI = 0.0595 to 0.782) and 0.842 (95% CI = 0.77 to 0.914), respectively, showing an even greater improvement of the AUC when the CRPv was considered (Figure 2).

**Table 5:** Comparison of different parameters between patients with bacterial and non-bacterial febrile illness presenting with a C-reactive protein concentration less than 100 mg/L

	**Variable**	**Duration of fever (hours)**	**CRP (mg/L)**	**CRP-velocity (mg/L/hour)**	**WBC (10^3^/ml^3^)**	**Neutrophils (10^3^/ml^3^)**
Bacterial (n = 59)	Mean	27.2	30.58	2.6	13.1	11.0
	Interquartile range	4.0 to 24.5	18.6 to 80.6	1.1 to 6.0	8.4 to 16.6	6.79 to 14.87

Non-bacterial (n = 63)	Mean	67.7	12.16	0.35	8.3	5.7
	Interquartile range	17 to 88	8.3 to 36.8	0.16 to 1.04	5.9 to 9.9	3.5 to 7.3

*P *value		< 0.001	< 0.001	< 0.001	< 0.001	< 0.001

CRP = C-reactive protein; WBC = white blood cells.

A cut-off value of 1.08 mg/L/hour (sensitivity 78%, specificity 77.8%) was chosen using the Youden index (= Sensitivity + Specificity - 1). Based on these observations, we defined a two-step model for the diagnosis of bacterial infection: patients with a CRP of 100 mg/L or higher, or patients with a CRP less than 100 mg/L and a CRPv of 1.08 mg/L/hour or higher, which demonstrated a sensitivity of 88% and a specificity of 70% for the diagnosis of bacterial infection as the origin of fever.

**Figure 3:**
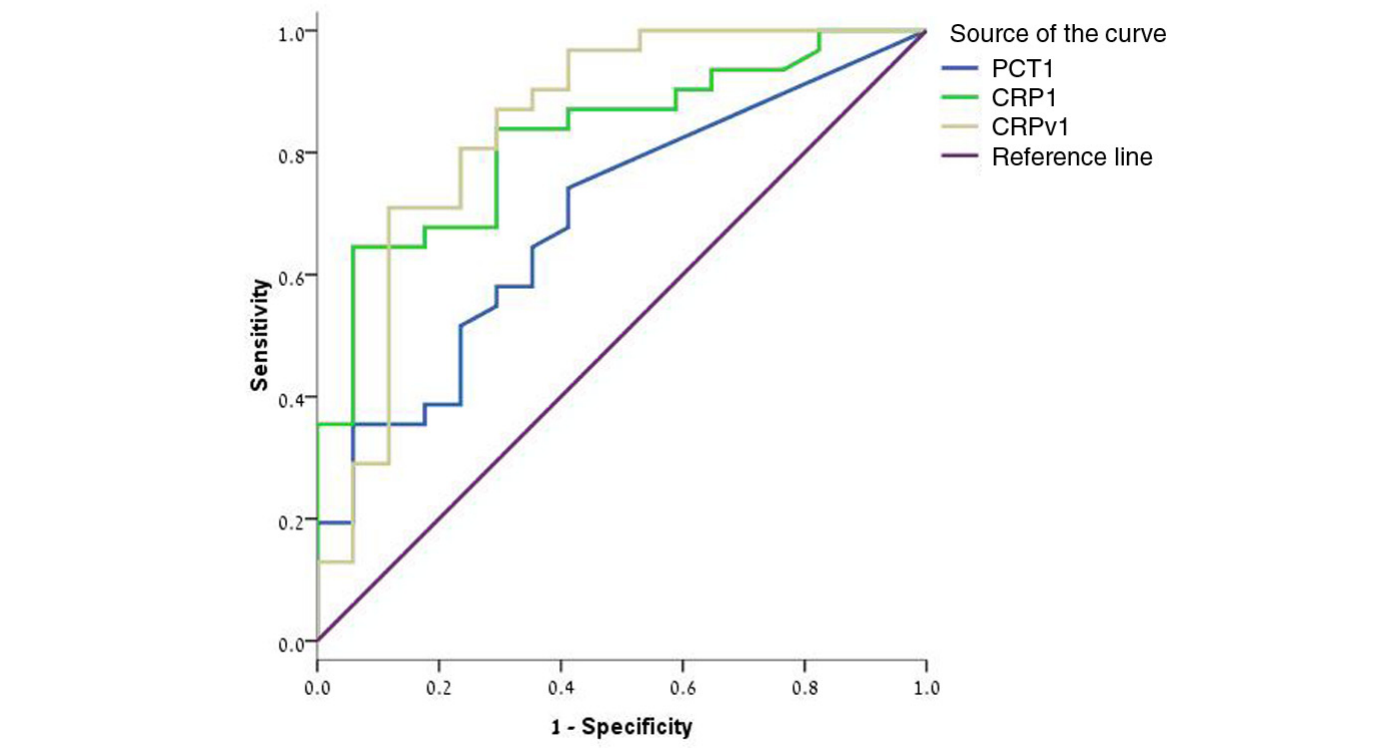
Receiver operator curve for C-reactive protein, C-reactive protein velocity and procalcitonin for the diagnosis of bacterial infection in a subgroup of 48 patients, 31 of whom had bacterial infections. CRP = C-reactive protein; CRPv = C-reactive protein velocity; PCT = procalcitonin.

PCT was also measured in a subgroup of 48 patients, 31 of whom had bacterial infections and 17 of whom had non-bacterial febrile illness. The median CRP, CRPv and PCT were significantly higher for the patients with bacterial infections than for the patients with non-bacterial febrile illness (Table 6). The efficacy of CRP measurements, CRPv and the PCT in differentiating between bacterial and non-bacterial febrile illness was evaluated by ROC analysis for this subgroup of patients. The AUC for CRP, CRPv and PCT were 0.824 (95% CI = 0.707 to 0.942), 0.844 (95% CI = 0.714 to 0.974) and 0.693 (95% CI = 0.54 to 0.846), respectively – significantly better for CRP and CRPv as indicated by the non overlapping 95% CIs (Figure 3).

**Table 6:** The median and interquartial range of C-reactive protein, C-reactive protein velocity and procalcitonin for the bacterial and non-bacterial groups in a subgroup of 48 patients

	Non-bacterial (n = 17)		Bacterial (n = 31)		*P *value
	Median	Interquartile range	Median	Interquartile range	
PCT (ng/ml)	0.05	0.05 to 0.24	0.21	0.05 to 0.53	0.025
CRP (mg/L)	29.0	15.5 to 52.1	99.9	41.3 to 143.7	< 0.001
CRPv (mg/L/hour)	0.44	0.26 to 1.32	2.67	1.39 to 5.16	< 0.001

CRP = C-reactive protein; CRPv = C-reactive protein velocity; PCT = procalcitonin.

## Discussion

The results of the current investigation demonstrated that it is possible to improve the differentiation between acute bacterial and non-bacterial febrile illnesses in the setting of the emergency room by using the parameter of CRPv instead of the absolute concentration of CRP alone. This diagnostic improvement proved to be significant and involved no additional cost due to the fact that it needed only the acquisition of additional information. This benefit, however, might be relevant only in patients for whom the time of fever onset is known.

To the best of our knowledge, the usefulness of a biomarker velocity, such as CRP, has not been tested in the context of differentiation between acute bacterial and non-bacterial febrile illnesses. Our results are therefore significant because they show the feasibility of using this diagnostic property of CRP in relation to the duration of the acute febrile disease.

The rationale for using the value of CRPv stems from the assumption that severe infections might be associated with a cytokine storm. In fact, cytokine storms are frequently seen in the context of acute and severe bacterial infections [13-17]. We hypothesized that a more rapid increment in the concentration of these cytokines might translate into a more rapid synthesis of the presently used biomarker, CRP.

The finding that patients with acute bacterial infections present with higher CRP concentrations than those who have non-bacterial febrile illnesses has been often shown in the past [1-8]. We now add the observation that patients with acute bacterial infections present to the emergency room earlier than those with acute non-bacterial infections. The relatively late arrival of individuals with acute non-bacterial febrile illness might be associated with a lower concentration of cytokines and therefore a lesser feeling of being unwell than individuals who have acute and severe bacterial infections.

Combining these two facts (higher CRP concentrations and earlier presentations), one can understand the behavior of the biomarker; namely, a steeper rise in patients with acute bacterial infections as opposed to a slower increment in those with non-bacterial febrile illnesses. In addition, the CRPv might have special relevance in individuals who present with concentrations that are not very high (i.e., less than 100 mg/L). Indeed, sensitivities and specificities of up to 90% have been shown for bacterial infections in individuals who present with CRP concentrations of 100 mg/L or more [2,11,12]. The improved results observed in our sub-analysis on patients with CRP concentrations of less than 100 mg/L support the notion that CRPv might be especially useful for them.

Different biomarkers have been previously tested for the purpose of differentiating between bacterial and non-bacterial febrile illnesses. These biomarkers include CRP [1-8], PCT [7,18-22], the absolute neutrophil count and IL-6 [5,6,16]. Although the populations in these studies were not similar to ours, the results reported in the literature were generally inferior to those we reported. Moreover, we measured PCT levels in a subgroup of 48 consecutive patients and found CRPv to be a better marker compared with PCT in distinguishing bacterial from non-bacterial febrile illnesses. Recent study on the combination of six different biomarkers (CRP, PCT, neutrophils, macrophage migration inhibitory factor, soluble urokinase-type plasminogen activator receptor and soluble triggering receptor expressed on myeloid cells-1) for the detection of bacterial versus non-bacterial febrile illness in patients with systemic inflammatory response syndrome reported an AUC of 0.88 [23]. In comparison, our newly defined parameter of CRPv showed similar efficacy without using any other test and without any further costs other than adding another simple question to history taking.

One limitation of the present study is that a clear bacterial etiology could not be obtained in all individuals in the bacterial infection group. This is especially relevant for the ones with pneumonia and gastroenteritis/colitis. In all cases we consulted infectious disease specialists. We agree that some cases of bacterial infections might have been viral and vice versa, but this limitation is known in similar studies. Another limitation is that every person has certain background inflammatory activity that is ignored in our calculations. However, this background inflammation is generally in the range of CRP of less than 10 mg/L [24], so this problem probably has a minor effect. Moreover, patients with a bacterial infection without clinically apparent fever were not included in this study. Hence, our conclusions are only relevant to patients with apparent fever. Finally, the main concern about the interpretation of the CRPv is its dependence on the time that has elapsed between the onset of fever and the measurement of CRP.

## Conclusions

In conclusion, we demonstrate that by adding the value of CRPv and not only looking at the absolute CRP concentration, it is possible to improve the differentiation between acute bacterial and non-bacterial febrile illnesses. CRPv is cost free and could be applied as a useful diagnostic tool to identify individuals with bacterial infection.

## Key messages

• CRPv distinguishes febrile bacterial infections from non-bacterial febrile illnesses better than CRP alone.

• CRPv distinguishes febrile bacterial infections from non-bacterial febrile illnesses better than CRP alone especially in patients with CRP levels less than 100 mg/L at presentation.

• CRPv correlates with other acute-phase proteins such as IL-1, IL-6, and TNF-α.

• CRPv is feasible in the setup of the emergency department.

## Abbreviations

AUC: area under the curve; BMI: body mass index; CI: confidence interval; CRP: C-reactive protein; CRPv: C-reactive protein velocity; IL: interleukine; PCT: procalcitonin; ROC: receiver operated curve; SD: standard deviation; TNF-α: tumor necrosis factor-α; WBC: white blood count.

## Competing interests

The authors declare that they have no competing interests.

## Authors' contributions

PH, SB, YP, DY, and GC participated in the study design and coordination. OR, RBA, MK and HS analysed the data. IZ, TR, and YO carried out the laboratory assays. YP, SB, and DJ drafted the manuscript.
